# Diagnostic value of dual-source, dual-energy computed tomography combined with the neutrophil-lymphocyte ratio for discriminating gastric signet ring cell from mixed signet ring cell and non-signet ring cell carcinomas

**DOI:** 10.1007/s00261-024-04286-9

**Published:** 2024-03-25

**Authors:** Qinxia Song, Xiangfa Wang, Juan Zhu, Hengfeng Shi

**Affiliations:** Department of radiology, Anqing Municipal Hospital, Anqing, 246000 Anhui province China

**Keywords:** Computed tomography, dual-source, Gastric adenocarcinoma subtypes

## Abstract

**Purpose:**

To explore the diagnostic value of dual-source computed tomography (DSCT) and neutrophil to lymphocyte ratio (NLR) for differentiating gastric signet ring cell carcinoma (SRC) from mixed SRC (mSRC) and non-SRC (nSRC).

**Methods:**

This retrospective study included patients with gastric adenocarcinoma who underwent DSCT between August 2019 and June 2021 at our Hospital. The iodine concentration in the venous phase (IC_vp_), standardized iodine concentration (NIC_VP_), and the slope of the energy spectrum curve (k_VP_) were extracted from DSCT data. NLR was determined from laboratory results. DSCT (including IC_VP_, NIC_VP_, and k_VP_) and combination (including DSCT model and NLR) models were established based on the multinomial logistic regression analysis. The receiver operator characteristic (ROC) curve and area under the curve (AUC) were used to evaluate the diagnostic value.

**Results:**

A total of 155 patients (SRC [*n* = 45, aged 61.22 ± 11.4 years], mSRC [*n* = 60, aged 61.09 ± 12.7 years], and nSRC [*n* = 50, aged 67.66 ± 8.76 years]) were included. There were significant differences in NLR, IC_VP_, NIC_VP_, and k_VP_ among the SRC, mSRC, and nSRC groups (all *P* < 0.001). The AUC of the combination model for SRC vs. mSRC + nSRC was 0.964 (95% CI: 0.923-1.000), with a sensitivity of 98.3% and a specificity of 86.7%, higher than with DSCT (AUC: 0.959, 95% CI: 0.919–0.998, sensitivity: 90.0%, specificity: 89.9%) or NLR (AUC: 0.670, 95% CI: 0.577–0.768, sensitivity: 62.2%, specificity: 61.8%).

**Conclusion:**

DSCT combined with NLR showed high diagnostic efficacy in differentiating SRC from mSRC and nSRC.

**Supplementary Information:**

The online version contains supplementary material available at 10.1007/s00261-024-04286-9.

## Introduction

Gastric adenocarcinoma is the fourth most common type of cancer and the second leading cause of cancer death in the world [1, 2]. According to the proportion of mucinous components, gastric adenocarcinoma can be divided into signet ring cell carcinoma (SRC), mixed signet ring cell carcinoma (mSRC), and non-signet ring cell carcinoma (nSRC) [3]. In recent decades, despite a decrease in the overall incidence of gastric adenocarcinoma, the incidence of SRC is constantly increasing [4, 5]. The prognosis of gastric adenocarcinoma containing signet ring cell components is poor, and the prognosis of mSRC (which shows stronger invasive ability than SRC) is significantly worse than SRC and ordinary gastric adenocarcinoma [3, 6]. Chen et al. [7] reported that adjuvant chemotherapy significantly improved the prognosis of patients with mSRC, while patients with SRC could not benefit from it. In addition, the response to perioperative radiotherapy and chemotherapy was also related to the proportion of signet ring cells in the tumors [8]. Therefore, identifying or predicting the pathological tumor type and the proportion of SRC as early as possible carries significant clinical implications.

Computed tomography (CT) imaging is widely used in diagnosing and staging gastric adenocarcinoma. Dual-source CT (DSCT) is a device based on mature multislice CT technology and has made a great breakthrough in time resolution [9]. The two detectors are placed at 90° and can decrease motion artifacts caused by the motion of arteries. In addition, the two tubes can be operated at different energy levels, increasing image quality and increasing the resolution of different tissues that could have the same appearance at a given energy value but a different appearance at another energy level [10]. Feature extraction based on intelligent segmentation algorithms by DSCT is fast and highly accurate [11, 12]. The normalized iodine concentration (NIC) in the arterial and venous phases positively correlates with microvessel density (MVD) [13, 14], reflecting the angiogenesis of different pathological subgroups of advanced gastric adenocarcinoma. The energy spectrum curve of DSCT used in the dual-energy mode helps evaluate the pathological type of gastric adenocarcinoma due to better tissue and structure differentiation [15].

Different cancers and cancer subtypes have different biology, and laboratory parameters can also be used to help discriminate among them. The neutrophil-to-lymphocyte ratio (NLR) is calculated as the ratio of neutrophils to lymphocytes in peripheral blood. The normal NLR values in adult, non-geriatric individuals in good health are between 0.70 and 0.78 and 3.00-3.53 [16, 17]. Recent studies revealed the diagnostic value of NLR in differentiating between benign and malignant lesions [18, 19]. The NLR is also a commonly used marker associated with short- and long-term outcomes in patients with gastric adenocarcinoma [20, 21]. A recent study revealed that the NLR could differentiate between two histological groups of gastric cancers (differentiated [tubular adenocarcinoma well-differentiated type, tubular adenocarcinoma moderately differentiated type, and papillary adenocarcinoma] vs. undifferentiated [poorly differentiated adenocarcinoma solid type, poorly differentiated adenocarcinoma solid type, and SRC]) [22]. The NLR predicts survival [23] or lymph node positivity [24] in patients with gastric SRC.

Although a previous study showed that SRC has higher iodine concentrations upon enhancement [25], no previous study examined the diagnostic value of DSCT combined with NLR in the differentiation of SRC, mSRC, and nSRC. Therefore, this study aimed to explore the diagnostic value of DSCT combined with NLR for distinguishing SRC from mSRC and nSRC.

## Methods

### Study design and patients

This retrospective study included patients with gastric adenocarcinoma who underwent DSCT between August 2019 and June 2021 at our Hospital. The inclusion criteria were (1) stage III-IV gastric adenocarcinoma confirmed by postoperative pathology, (2) underwent DSCT within 1 week of surgery, (3) well-filled stomach and clear images, and (4) complete data (i.e., a complete dataset for the variables presented in the Tables). The exclusion criteria were (1) other malignant tumors, (2) incomplete clinical or imaging data, or (3) neoadjuvant chemotherapy or other treatments before DSCT. This study was approved by the ethics committee of our Hospital (approval 2023 (17)). The requirement for individual informed consent was waived by the committee because of the retrospective nature of the study. SRC was defined as the presence of a predominant component (> 50%) of isolated carcinoma cells containing mucin. mSRC was defined as adenocarcinoma with a minor component (10-50%) of isolated carcinoma cells containing mucin. nSRC was defined as only adenocarcinoma [3]. The initial pathological diagnosis was based on the diagnosis in the electronic patient chart system. To ensure accuracy, all slides underwent reanalysis by a pathologist specialized in gastric cancer, who visually confirmed the proportion of signet ring cells at high magnification.

### Image acquisition

The patients were required to fast for 6 h and drink 800–1000 mL of warm water before the scan. The patient was in a supine position, and the scan was performed using a Siemens Definition Flash CT machine, covering the area from the diaphragm to the sacroiliac joint. The routine scan parameters during the study period were collimation of 128 × 0.6 mm, reconstruction with the B30f kernel, and fusion coefficient of 0.5. Tube voltage was set at 100/140 kV. The contrast agent, Iohexol (320 mg I/mL), was injected at 2.5-3.5mL/s using a high-pressure injector, with a total volume of 60 mL mixed with 20 mL of saline solution. Arterial phase scanning began when the CT value of the abdominal aorta reached 100 HU, followed by a 30-s delay for the venous phase. Data was reconstructed with a thickness of 1 mm and transferred to the Syngo Via workstation (Siemens Healthcare, Erlangen, Germany).

### Data collection and imaging analysis

The demographic and imaging data were collected from the medical records of the patients. The DSCT parameters were measured according to medical imaging records. In this study, blood cell counts, including neutrophils and lymphocytes, were derived from the most recent preoperative complete blood counts in the electronic medical record system. The blood tests were performed during the preoperative workup, generally 5 days before surgery.

Using a Siemens Syngo Via workstation (Siemens Healthcare, Erlangen, Germany), the thin-layer images of venous phases were analyzed using the liver virtual non-contrast (VNC) mode, which can generate the iodine map. A region of interest (ROI) was outlined in the abdominal aorta at the largest level of the lesion. The ROI was located in the center of the abdominal aorta, accounting for more than 1/2 of the cross-sectional area. Then, circular ROIs were outlined in the visibly contrast-enhancing border of the tumors, avoiding the visible blood vessels, calcification, and necrosis areas, with a diameter greater than 1/2 of the thickness of the lesion. IC_lesion_ was calculated as the iodine concentrations of the ROI. IC_aorta_ was the iodine concentration of the abdominal aorta. In the iodine density image derived from the iodine/water-based material decomposition image, the concentration of iodine in lesions was measured in the venous phase (IC_VP_). Besides, the normalized iodine concentration (NIC_VP_) was calculated as IC_lesion_/IC_aorta_ in the venous phase. Using the dual energy software, the system automatically reconstructed 70-keV single energy images in the venous phase. Then, by selecting the monoenergetic program to reconstruct the 40-keV and 100-keV single energy images in the venous phase, the CT value of the ROI was measured at the same level, position, and size in the 40-keV and 100-keV images, and the k_VP_ was calculated according to the formula k_VP_=CT_40 keV_-CT_100 keV_/100 − 40. The ROIs were as large as possible and were measured in axial images for two or three continuous layers. The image characteristics were measured three times by a senior physician (with 17 years of working experience) who was blinded to the pathology results; the average data were taken. A second physician also measured the image characteristics three times. The internal consistency test revealed κ = 0.823 (*P* < 0.001). The typical cases for imaging analysis are shown in Supplementary Figures S1–S3.

### Statistical analysis

All data were analyzed using SPSS 26.0 (IBM, Armonk, NY, USA). The continuous data with a normal distribution were expressed as means ± standard deviations and analyzed using one-way ANOVA and the Bonferroni post hoc test. The categorical data were presented as n (%) and analyzed using the chi-square test. The prediction models were established based on multinomial logistic regression analysis. The DSCT model for SRC vs. mSRC + nSRC included IC_VP_, NIC_VP_, and k_VP_. The combination model included the DSCT model and NLR. Receiver operating characteristics (ROC) curves were created to calculate the area under the curve (AUC) and evaluate the diagnostic efficiency. DeLong’s test was used to compare the AUCs. A two-sided *P* < 0.05 was considered statistically significant.

## Results

A total of 155 patients (89 males, aged 63.2 ± 11.5 years) were included and divided into the SRC (*n* = 45, 22 males), mSRC (*n* = 60, 34 males), and nSRC (*n* = 50, 33 males) groups. There were no significant differences in age, sex, and tumor location among the three groups (all *P* > 0.05) (Table 1).

**Table 1:** Characteristics of the patients

Characteristics	SRC (*n* = 45)	mSRC (*n* = 60)	nSRC (*n* = 50)	*P*
Age	61.22 ± 11.4	61.09 ± 12.7	67.66 ± 8.76	0.275
Sex				0.065
Male	22	34	33	
Female	23	26	17	
NLR	3.77 ± 2.34	2.88 ± 1.61 ^a^	2.09 ± 0.89 ^a^	< 0.001
Tumor location				0.155
Fundus	7	19	20	
Body	12	24	10	
Antrum	26	17	20	

SRC: signet ring cell carcinoma; mSRC: mixed signet ring cell carcinoma; nSRC: non-signet ring cell carcinoma; NLR: neutrophil-to-lymphocyte ratio

^a^*P* < 0.001 vs. the SRC group

The IC_VP_ were significantly different among patients in the SRC, mSRC, and nSRC groups (2.76 ± 0.11 vs. 2.15 ± 0.07 vs. 2.02 ± 0.09, *P* < 0.001), as well as NIC_VP_ (0.57 ± 0.03 vs. 0.46 ± 0.15 vs. 0.45 ± 0.02, *P* < 0.001) and k_VP_ (3.56 ± 0.06 vs. 3.15 ± 0.07 vs. 3.03 ± 0.09, *P* < 0.001). Besides, the NLR was also significantly different among patients in the SRC, mSRC, and nSRC groups (0.57 ± 0.03 vs. 0.46 ± 0.15 vs. 0.45 ± 0.02, *P* < 0.001) (Table 2).

**Table 2:** Energy spectrum characteristics and NLR

Characteristics	SRC (*n* = 45)	mSRC (*n* = 60)	nSRC (*n* = 50)	*P*
IC_VP_	2.76 ± 0.11	2.15 ± 0.07 ^a^	2.02 ± 0.09 ^a^	< 0.001
NIC_VP_	0.57 ± 0.03	0.46 ± 0.15 ^b^	0.45 ± 0.02 ^c^	< 0.001
k_VP_	3.56 ± 0.06	3.15 ± 0.07 ^a^	3.03 ± 0.09 ^a^	< 0.001
NLR	3.77 ± 2.34	2.88 ± 1.61	2.09 ± 0.89	< 0.001

SRC: signet ring cell carcinoma; mSRC: mixed signet ring cell carcinoma; nSRC: non-signet ring cell carcinoma; IC_VP_: iodine concentration in venous phase; NIC_VP_: standardized iodine concentration in venous phase; k_VP_: slope of energy spectrum curve in venous phase; NLR: neutrophil-to-lymphocyte ratio

^a^*P* < 0.001 vs. the SRC group

^b^*P* = 0.001 vs. the SRC group

^c^*P* = 0.002 vs. the SRC group

**Fig. 1:**
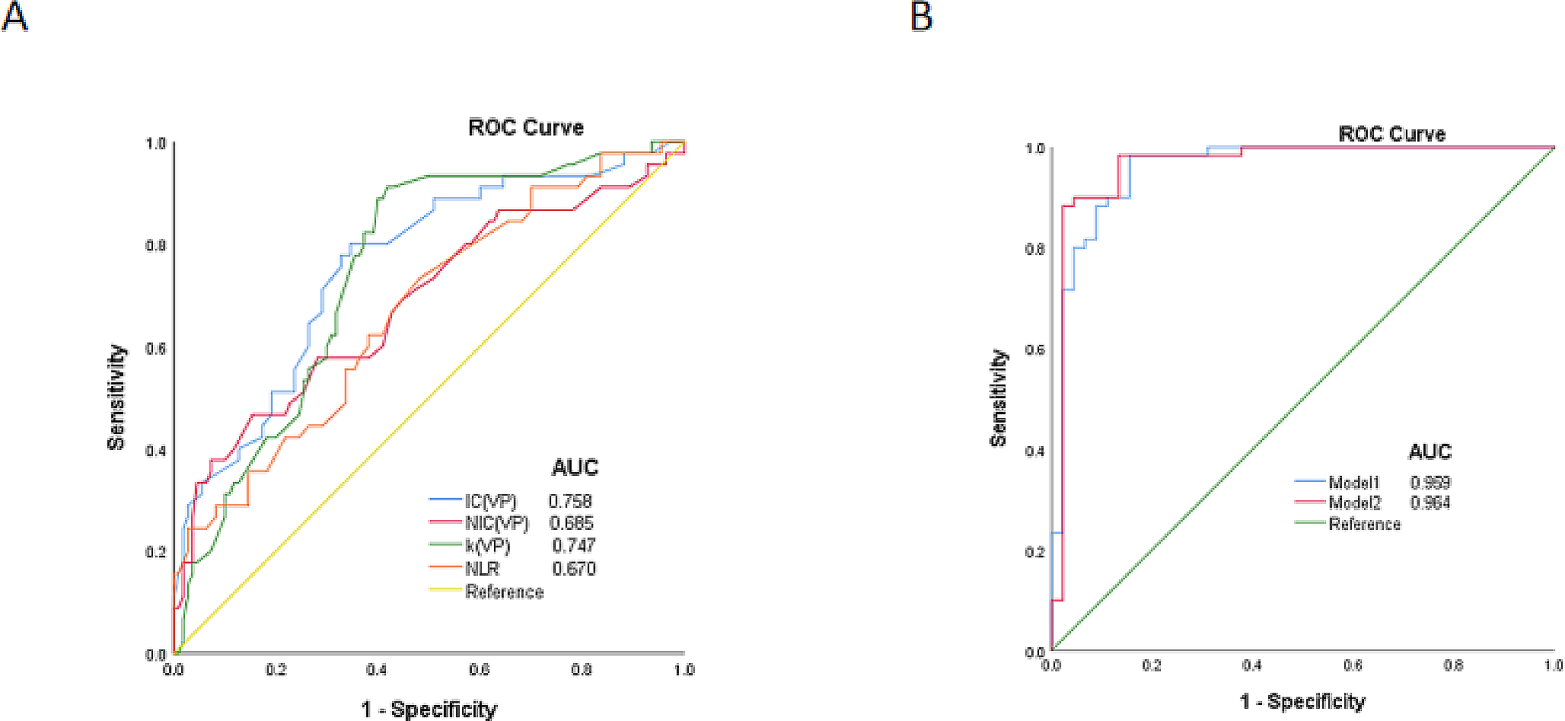
The Receiver operating characteristics curve analyses for diagnostic value. (**A**) Diagnostic value of iodine concentration (IC), standardized iodine concentration (NIC), the slope of energy spectrum curve (k), and neutrophil-to-lymphocyte ratio (NLR). (**B**) The diagnostic value of dual-source computed tomography (DSCT) model (model1, IC + NIC + k) and combination model (model 2, DSCT model and NLR).

The AUC of IC_VP_, NIC_VP_, k_VP_, and NLR for the differential diagnosis of SRC vs. mSRC + nSRC were 0.758 (95% CI: 0.674–0.842), 0.685 (95% CI: 0.587–0.784), 0.747 (95% CI: 0.666–0.827), and 0.670 (95% CI: 0.577–0.768), respectively. The AUC of the DSCT model was 0.959 (95% CI: 0.919–0.998), with 90.0% sensitivity and 88.9% specificity. The AUC of the combination model was 0.964 (95% CI: 0.923-1.000), with 98.3% sensitivity and 86.7% specificity (Tables 3 and Fig. 1).

**Table 3:** Diagnosis performance of parameters and models for SRC vs. mSRC + nSRC

Characteristics	Sensitivity (%)	Specificity (%)	AUC	cut-off	SE	95%CI
IC_VP_	77.8	67.3	0.758	2.32	0.0494	0.674–0.842
NIC_VP_	68.9	65.0	0.685	0.49	0.0555	0.587–0.784
k_VP_	88.9	60.0	0.747	3.29	0.0493	0.666–0.827
NLR	62.2	61.8	0.670	2.33	0.0557	0.577–0.768
DSCT model	90.0	88.9	0.959	0.59	0.0364	0.919–0.998
Combination model	98.3	86.7	0.964	0.54	0.0364	0.923-1.000

The DeLong test was used to compare the AUC between parameters. IC_VP_: iodine concentration in venous phase; NIC_VP_: standardized iodine concentration in venous phase; k_VP_: slope of energy spectrum curve in venous phase; NLR: neutrophil-to-lymphocyte ratio; AUC: area under the receiver operating characteristic curve. DSCT, dual-source computed tomography. The DSCT model included IC_VP_, NIC_VP_, and k_VP_. And the combination model included the DSCT model and NLR.

## Discussion

Recently, DSCT was applied for the staging of gastric cancer [26], and a complete blood count is a basic routine examination. Using data from DSCT and complete blood count is practical and noninvasive, although histological analysis by biopsy of the gastric lesion before any treatment is indispensable for clinical management [27]. It is reported that among gastric carcinoma types, SRC shows less aggressive biologic characteristics, and mixed-SRC shows more aggressive characteristics, which must be considered for planning therapies [3]. Thus, DSCT combined with NLR as a noninvasive method could be used to differentiate SRC in early gastric cancer, which may be helpful in deciding on a specific cancer treatment. The findings might provide evidence for the application of DSCT in the noninvasive differential diagnosis of gastric adenocarcinoma subtypes.

In this study, IC_VP_ and NIC_VP_, as indicators of angiogenesis and MVD [14], were significantly higher in the SRC group than in the mSRC and nSRC groups, but there were no significant differences in IC_VP_ and NIC_VP_ between the mSRC and nSRC groups. The reason may be that SRC has abundant neovascularization and high permeability, while mSRC has low MVD [28]. The results also showed that the k_VP_ of the SRC group was significantly higher than in the mSRC and nSRC groups, which may be related to the relatively rich blood supply of SRC tumors [29], leading to higher iodine contrast agent after enhancement and higher CT values when at lower keV.

The possibility of using the NLR to differentiate between benign and malignant conditions has been shown for gallbladder [18, 30] and adrenal lesions [19]. On the other hand, studies of the NLR in gastric cancer are rarer. A study showed that the inflammatory response and preoperative NLR were prognostic markers in patients with gastric adenocarcinoma [31]. Two other studies showed that the NLR was associated with prognosis [23] and lymph node positivity [24] in patients with gastric SRC. Regarding the diagnostic value of NLR for SRC, a recent study revealed that the NLR could differentiate between differentiated (not including SRC) vs. undifferentiated (including SRC) gastric cancers [22]. The present study appears to be the first to directly compare the NLR among SRC, mSRC, and nSRC, showing that the NLR was significantly different among the three subtypes. The normal NLR values in adult, non-geriatric individuals in good health are between 0.70 and 0.78 and 3.00-3.53 [16, 17], but variations in NLR can be observed among patients, even if they are within the normal range. Of note, the optimal cut-off value determined in the ROC analysis was 2.33 in the present study.

In the present study, the AUCs of the individual DSCT parameters and NLR were 0.670–0.758. The AUC values of the individual parameters were lower than that of CT-based radiomics nomograms for identifying SRC (Lauren nomogram, AUC = 0.841–0.895; SRCC nomogram, AUC = 0.845–0.918) [32], suggesting that the single indexes had a relatively poor diagnostic performance. On the other hand, the AUC of the combination of all four parameters was 0.964 for differentiating SRC from mSRC and nSRC. That combination had a higher AUC than in the radiomics study by Chen et al. [32]. Hence, the results support the use of that model for differentiating SRC from mSRC and nSRC, but external validity will have to be examined.

There were several limitations in this study. First, this study was conducted in a single center, limiting the sample size. The small size limits the reliability of the evaluation, and the single center limits the generalizability of the results. Second, this study was retrospective, which may cause data bias, limiting the analyzable data to those available in the patient charts and preventing the determination of any cause-to-effect relationships [33]. Third, multivariable analyses can be clinically invalid since they are based on the included variables, which depend upon the available variables. Hence, different studies that collected different variables can reach different conclusions. Fourth, the blood tests for NLR were generally performed 5 days before surgery, but it was a retrospective study, and it is possible that the timing might be different for some patients. The next step would be to increase the sample size and conduct prospective studies to continue exploring the diagnostic value of DSCT combined with the NLR for distinguishing SRC from mSRC and nSRC. In addition, T2WI imaging can help determine the mucus component in cancers [34, 35], but the present study only included DSCT data, not MRI. Nevertheless, future studies could investigate a combination of FSCT and MRI parameters for distinguishing SRC, mSRC, and nSRC.

In conclusion, DSCT combined with NLR showed high diagnostic efficacy to differentiate SRC from mSRC and nSRC. However, the result still requires prospective studies to be confirmed in the future. Nevertheless, the model could eventually be used to help guide patient management.

### Electronic supplementary material

Below is the link to the electronic supplementary material.

**Supplementary Fig. 1.** 82-year-old woman with gastric signet ring cell carcinoma of (SRC).(A)Iodine concentration in venous phase(ICvp),IC = 3.2 mg/ml;standardized iodine concentration(NIC_VP_),NIC = 0.80;(B)the slope of energy spectrum curve(k_VP_),k = CT40keV-CT100keV/100 − 40 = 300.6–75.5/60 = 3.75

**Supplementary Fig. 2.** 57-year-old man with gastric mixed signet ring cell carcinoma(mSRC).(A)Iodine concentration in venous phase(IC_VP_),IC = 2.4 mg/ml;standardized iodine concentration(NIC_VP_),NIC = 0.44;(B)the slope of energy spectrum curve(k_VP_),k = CT40keV-CT100keV/100 − 40 = 245.7–69.9/60 = 2.93

**Supplementary Fig. 3.** 80-year-old woman with gastric non signet ring cell carcinoma(nSRC).(A)Iodine concentration in venous phase(ICvp),IC = 1.6 mg/ml;standardized iodine concentration(NICVP),NIC = 0.43;(B)the slope of energy spectrum curve(kVP),k = CT40keV-CT100keV/100 − 40 = 202.5–67.2/60 = 2.26

## Data Availability

All data generated or analysed during this study are included in this published article.

## References

[1] Topi S, Santacroce L, Bottalico L et al (2020) Gastric Cancer in History: A Perspective Interdisciplinary Study. Cancers (Basel) 12. 10.3390/cancers12020264 10.3390/cancers12020264 PMC7072612 31978985

[2] Ilson DH (2021) Advances in the treatment of gastric cancer: 2020–2021. Curr Opin Gastroenterol 37:615–618. 10.1097/mog.0000000000000776 34456227 10.1097/MOG.0000000000000776 PMC9585687

[3] Huh CW, Jung DH, Kim JH et al (2013) Signet ring cell mixed histology may show more aggressive behavior than other histologies in early gastric cancer. J Surg Oncol 107:124–129. 10.1002/jso.23261 22991272 10.1002/jso.23261

[4] Wei Q, Gao Y, Qi C et al (2021) Clinicopathological Characteristics and Prognosis of Signet Ring Gastric Cancer: A Population-Based Study. Front Oncol 11:580545. 10.3389/fonc.2021.580545 34490073 10.3389/fonc.2021.580545 PMC8418067

[5] Koseki Y, Hatakeyama K, Terashima M et al (2023) Molecular profile of poorly cohesive gastric carcinoma with special reference to survival. Gastric Cancer. 10.1007/s10120-023-01390-5 37036539 10.1007/s10120-023-01390-5

[6] Dong X, Sun G, Qu H, He Q, Hao Z (2021) Prognostic Significance of Signet-Ring Cell Components in Patients With Gastric Carcinoma of Different Stages. Front Surg 8:642468. 10.3389/fsurg.2021.642468 34336913 10.3389/fsurg.2021.642468 PMC8319562

[7] Chen L, Shi Y, Yuan J et al (2014) Evaluation of docetaxel- and oxaliplatin-based adjuvant chemotherapy in postgastrectomy gastric cancer patients reveals obvious survival benefits in docetaxel-treated mixed signet ring cell carcinoma patients. Med Oncol 31:159. 10.1007/s12032-014-0159-5 25119501 10.1007/s12032-014-0159-5

[8] Charalampakis N, Nogueras González GM, Elimova E et al (2016) The Proportion of Signet Ring Cell Component in Patients with Localized Gastric Adenocarcinoma Correlates with the Degree of Response to Pre-Operative Chemoradiation. Oncology 90:239–247. 10.1159/000443506 27046280 10.1159/000443506 PMC4870109

[9] Chen Q, Wang X, Ding R, Wang Z (2021) Intelligent Algorithm-Based CT Imaging for Evaluation of Efficacy of Docetaxel Combined with Fluorouracil on Patients with Gastric Cancer. J Healthc Eng 2021:1040374. 10.1155/2021/1040374 34659676 10.1155/2021/1040374 PMC8514889

[10] Schmidt B, Flohr T (2020) Principles and applications of dual source CT. Phys Med 79:36–46. 10.1016/j.ejmp.2020.10.014 33115699 10.1016/j.ejmp.2020.10.014

[11] Tirumani SH, Shinagare AB, O’Neill AC, Nishino M, Rosenthal MH, Ramaiya NH (2016) Accuracy and feasibility of estimated tumour volumetry in primary gastric gastrointestinal stromal tumours: validation using semiautomated technique in 127 patients. Eur Radiol 26:286–295. 10.1007/s00330-015-3829-6 25991487 10.1007/s00330-015-3829-6 PMC4654722

[12] Han QY, Zhang X, Zhang JG et al (2022) Pre-operative neutrophil-to-lymphocyte ratio is an independent prognostic factor in patients with gastric cancer. Int Immunopharmacol 113:109371. 10.1016/j.intimp.2022.109371 36279674 10.1016/j.intimp.2022.109371

[13] Chen XH, Ren K, Liang P, Chai YR, Chen KS, Gao JB (2017) Spectral computed tomography in advanced gastric cancer: Can iodine concentration non-invasively assess angiogenesis? World J Gastroenterol 23:1666–1675. 10.3748/wjg.v23.i9.1666 28321168 10.3748/wjg.v23.i9.1666 PMC5340819

[14] Liang P, Ren XC, Gao JB, Chen KS, Xu X (2017) Iodine Concentration in Spectral CT: Assessment of Prognostic Determinants in Patients With Gastric Adenocarcinoma. AJR Am J Roentgenol 209:1033–1038. 10.2214/ajr.16.16895 28871809 10.2214/AJR.16.16895

[15] Lu Z, Wu S, Yan C, Chen J, Li Y (2021) Clinical value of energy spectrum curves of dual-energy computer tomography may help to predict pathological grading of gastric adenocarcinoma. Transl Cancer Res 10:1–9. 10.21037/tcr-20-1269 35116234 10.21037/tcr-20-1269 PMC8797754

[16] Forget P, Khalifa C, Defour JP, Latinne D, Van Pel MC, De Kock M (2017) What is the normal value of the neutrophil-to-lymphocyte ratio? BMC Res Notes 10:12. 10.1186/s13104-016-2335-5 28057051 10.1186/s13104-016-2335-5 PMC5217256

[17] Zahorec R (2021) Neutrophil-to-lymphocyte ratio, past, present and future perspectives. Bratisl Lek Listy 122:474–488. 10.4149/BLL_2021_078 34161115 10.4149/BLL_2021_078

[18] Kucuk S, Mızrak S (2021) Diagnostic Value of Inflammatory Factors in Patients with Gallbladder Cancer, Dysplasia, and Cholecystitis. Cancer Control 28:10732748211033746. 10.1177/10732748211033746 34348499 10.1177/10732748211033746 PMC8358487

[19] Sisman P, Bicer B, Gul OO et al (2020) May hemocytometer parameters be a biomarker in distinguishing between adrenal adenomas and carcinomas and in prognosis of adrenocortical carcinomas? Acta Clin Croat 59:439–444. 10.20471/acc.2020.59.03.07 34177053 10.20471/acc.2020.59.03.07 PMC8212649

[20] Miyamoto R, Inagawa S, Sano N, Tadano S, Adachi S, Yamamoto M (2018) The neutrophil-to-lymphocyte ratio (NLR) predicts short-term and long-term outcomes in gastric cancer patients. Eur J Surg Oncol 44:607–612. 10.1016/j.ejso.2018.02.003 29478743 10.1016/j.ejso.2018.02.003

[21] Du R, Ming J, Geng J et al (2022) Establishment of prognostic models for adenocarcinoma of oesophagogastric junction patients with neoadjuvant chemoradiotherapy: a real-world study. Radiat Oncol 17:45. 10.1186/s13014-022-02016-3 35241109 10.1186/s13014-022-02016-3 PMC8896317

[22] Yasui S, Takata T, Kamitani Y et al (2021) Neutrophil-to-Lymphocyte Ratio Is a Useful Marker for Predicting Histological Types of Early Gastric Cancer. J Clin Med 10. 10.3390/jcm10040791 10.3390/jcm10040791 PMC7920243 33669317

[23] Yang S, Li S (2022) Development of prognostic predictive model with neutrophil-lymphocyte ratio (NLR) in patients with gastric signet ring carcinoma. Medicine (Baltimore) 101:e28043. 10.1097/MD.0000000000028043 35029873 10.1097/MD.0000000000028043 PMC8735796

[24] Zhang LX, Wei ZJ, Xu AM, Zang JH (2018) Can the neutrophil-lymphocyte ratio and platelet-lymphocyte ratio be beneficial in predicting lymph node metastasis and promising prognostic markers of gastric cancer patients? Tumor maker retrospective study. Int J Surg 56:320–327. 10.1016/j.ijsu.2018.06.037 29969732 10.1016/j.ijsu.2018.06.037

[25] Chen J, Cai R, Ren G et al (2018) Differences in clinicopathological characteristics and computed tomography findings between signet ring cell carcinoma and nonsignet ring cell carcinoma in early and advanced gastric cancer. Cancer Med 7:1160–1169. 10.1002/cam4.1417 29533002 10.1002/cam4.1417 PMC5911613

[26] Xie ZY, Chai RM, Ding GC, Liu Y, Ren K (2018) T and N Staging of Gastric Cancer Using Dual-Source Computed Tomography. Gastroenterol Res Pract 2018:5015202. 10.1155/2018/5015202 30622560 10.1155/2018/5015202 PMC6304930

[27] Efared B, Kadi M, Tahiri L et al (2020) Gastric Signet Ring Cell Carcinoma: A Comparative Analysis of Clinicopathologic Features. Cancer Control 27:1073274820976596. 10.1177/1073274820976596 33269609 10.1177/1073274820976596 PMC8480344

[28] Stojanovic D, Milenkovic SM, Mitrovic N et al (2018) Clinical significance of neoangiogenesis and index of proliferation in the signet ring type of gastric adenocarcinoma. J buon 23:992–1003. 30358204

[29] Ji X, Yang Q, Qin H, Zhou J, Liu W (2019) Tumor blood supply may predict neoadjuvant chemotherapy response and survival in patients with gastric cancer. J Int Med Res 47:2524–2532. 10.1177/0300060519845491 31039658 10.1177/0300060519845491 PMC6567713

[30] Zhang J, Wu Y, Feng Y, Fu J, Jia N (2023) The value of CT findings combined with inflammatory indicators for preoperative differentiation of benign and malignant gallbladder polypoid lesions. World J Surg Oncol 21:51. 10.1186/s12957-023-02941-x 36803518 10.1186/s12957-023-02941-x PMC9938612

[31] Chen L, Chen Y, Zhang L et al (2021) In Gastric Cancer Patients Receiving Neoadjuvant Chemotherapy Systemic Inflammation Response Index is a Useful Prognostic Indicator. Pathol Oncol Res 27:1609811. 10.3389/pore.2021.1609811 34712105 10.3389/pore.2021.1609811 PMC8546636

[32] Chen T, Wu J, Cui C et al (2022) CT-based radiomics nomograms for preoperative prediction of diffuse-type and signet ring cell gastric cancer: a multicenter development and validation cohort. J Transl Med 20:38. 10.1186/s12967-022-03232-x 35073917 10.1186/s12967-022-03232-x PMC8785479

[33] Talari K, Goyal M (2020) Retrospective studies - utility and caveats. J R Coll Physicians Edinb 50:398–402. 10.4997/jrcpe.2020.409 33469615 10.4997/JRCPE.2020.409

[34] Huang A, Yang Y, Shi JY et al (2021) Mucinous adenocarcinoma: A unique clinicopathological subtype in colorectal cancer. World J Gastrointest Surg 13:1567–1583. 10.4240/wjgs.v13.i12.1567 35070064 10.4240/wjgs.v13.i12.1567 PMC8727185

[35] Cao W, Wu L, Zhao Y et al (2020) A New MRI-Defined Biomarker for Rectal Mucinous Adenocarcinoma: Mucin Pool Patterns in Determining the Efficacy of Neoadjuvant Therapy. Front Oncol 10:1425. 10.3389/fonc.2020.01425 32974154 10.3389/fonc.2020.01425 PMC7468516

