# Supplementary figures and images for: Diagnostic value of dual-source, dual-energy computed tomography combined with the neutrophil-lymphocyte ratio for discriminating gastric signet ring cell from mixed signet ring cell and non-signet ring cell carcinomas
Source: Abdom Radiol (NY). 2024 Mar 25;49(9):2996–3002. doi: 10.1007/s00261-024-04286-9 (PMC11335798; doi:10.1007/s00261-024-04286-9)

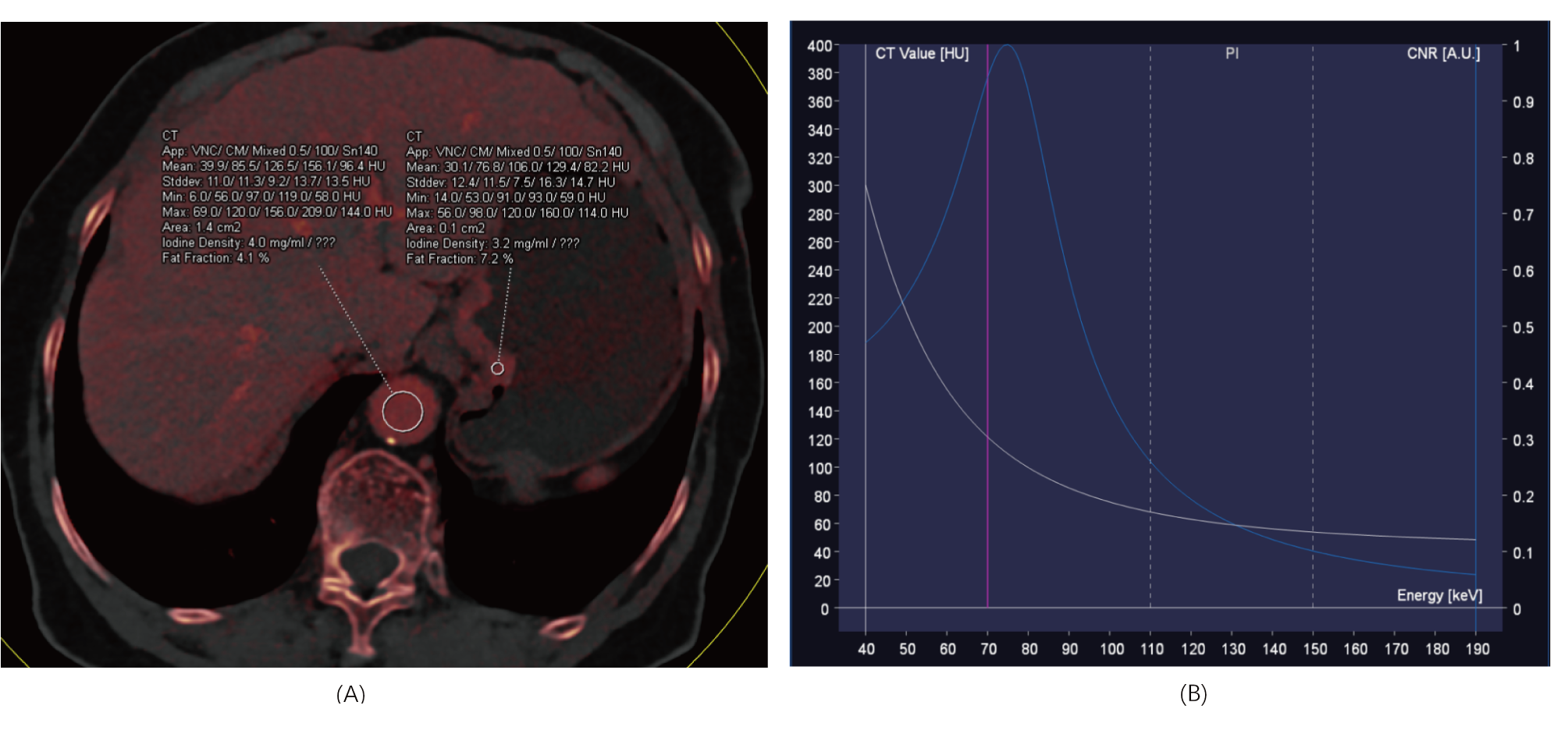

Supplement: Supplementary file 1 — Supplementary Fig. 1. 82-year-old woman with gastric signet ring cell carcinoma of (SRC).(A)Iodine concentration in venous phase(ICvp),IC = 3.2 mg/ml;standardized iodine concentration(NICVP),NIC = 0.80;(B)the slope of energy spectrum curve(kVP),k = CT40keV-CT100keV/100 − 40 = 300.6–75.5/60 = 3.75 [file 261_2024_4286_MOESM1_ESM.tif]

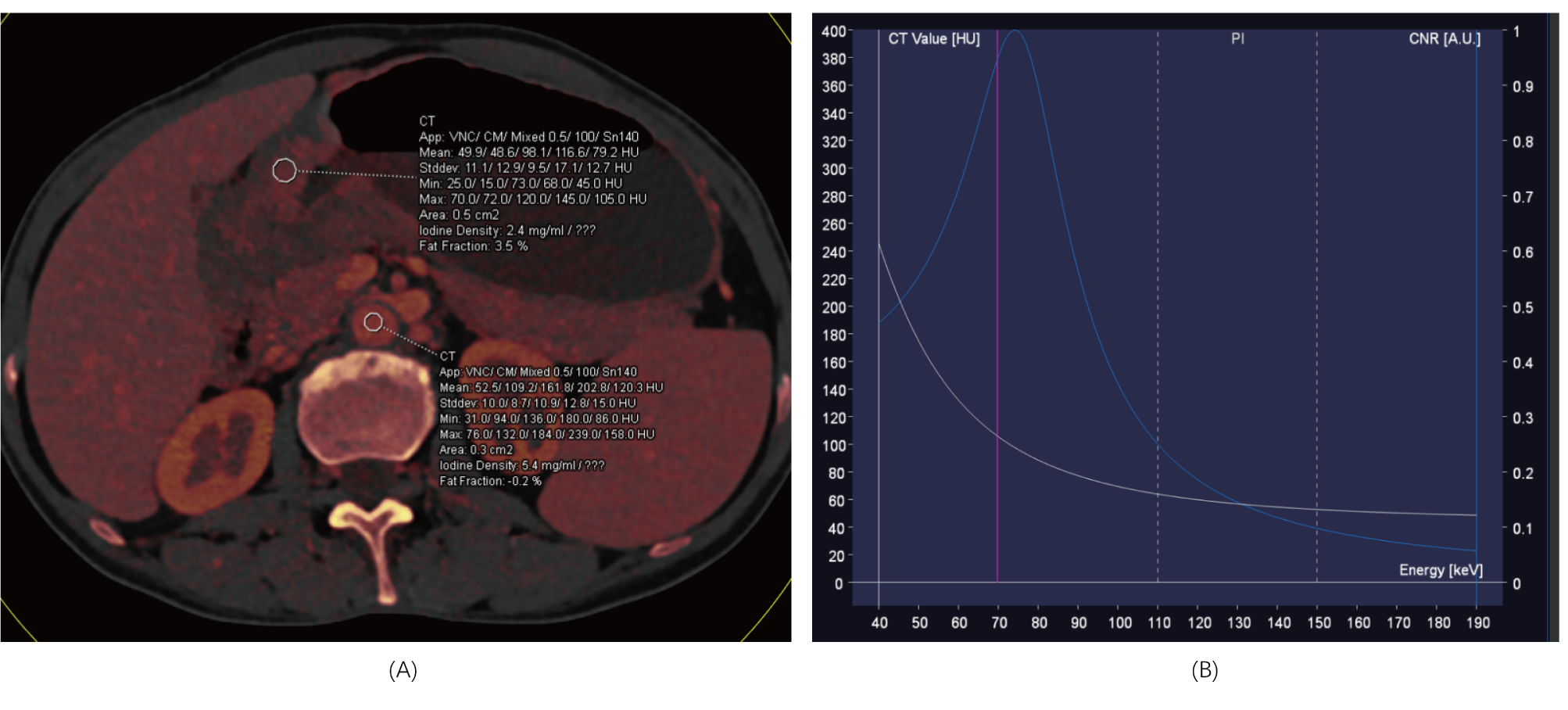

Supplement: Supplementary file 2 — Supplementary Fig. 2. 57-year-old man with gastric mixed signet ring cell carcinoma(mSRC).(A)Iodine concentration in venous phase(ICVP),IC = 2.4 mg/ml;standardized iodine concentration(NICVP),NIC = 0.44;(B)the slope of energy spectrum curve(kVP),k = CT40keV-CT100keV/100 − 40 = 245.7–69.9/60 = 2.93 [file 261_2024_4286_MOESM2_ESM.tif]

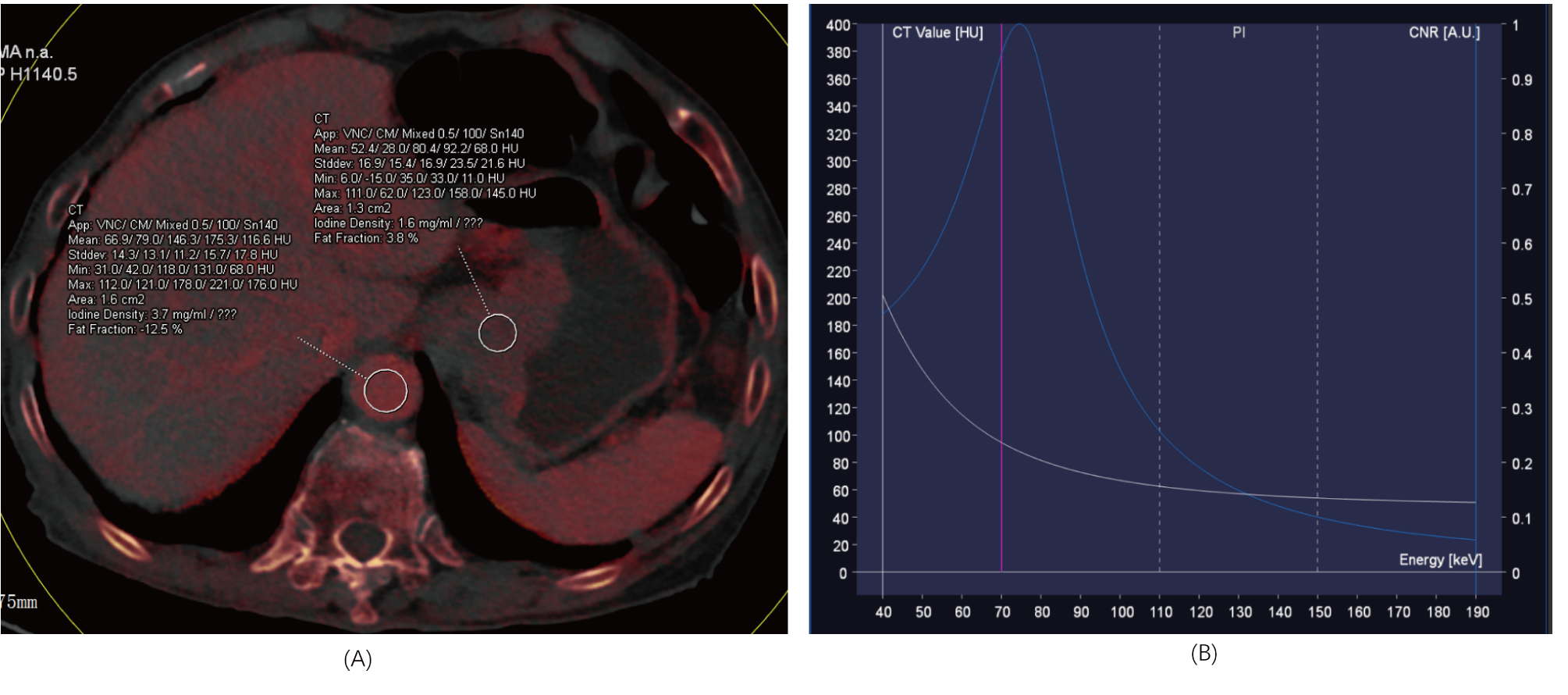

Supplement: Supplementary file 3 — Supplementary Fig. 3. 80-year-old woman with gastric non signet ring cell carcinoma(nSRC).(A)Iodine concentration in venous phase(ICvp),IC = 1.6 mg/ml;standardized iodine concentration(NICVP),NIC = 0.43;(B)the slope of energy spectrum curve(kVP),k = CT40keV-CT100keV/100 − 40 = 202.5–67.2/60 = 2.26 [file 261_2024_4286_MOESM3_ESM.tif]
